# Therapeutic effects of a novel synthetic α-secretase

**DOI:** 10.3389/fnagi.2024.1383905

**Published:** 2024-06-07

**Authors:** Sung Bin Kim, Bo-Ram Mun, Sung Yoon Kim, Muthukumar Elangovan, Euy Jun Park, Won-Seok Choi, Woo Jin Park

**Affiliations:** ^1^School of Life Sciences, Gwangju Institute of Science and Technology, Gwangju, Republic of Korea; ^2^School of Biological Sciences and Technology, Chonnam National University, Gwangju, Republic of Korea

**Keywords:** Alzheimer’s disease, amyloid-β clearance, amyloid-β degrading enzyme, synthetic α-secretase, nuclear inclusion a

## Abstract

Excessive accumulation of amyloid-β (Aβ) has been associated with the pathogenesis of Alzheimer’s disease (AD). Clinical studies have further proven that elimination of Aβ can be a viable therapeutic option. In the current study, we conceptualized a fusion membrane protein, referred to as synthetic α-secretase (SAS), that can cleave amyloid precursor protein (APP) and Aβ specifically at the α-site. In mammalian cells, SAS indeed cleaved APP and Aβ at the α-site. Overexpression of SAS in the hippocampus was achieved by direct injection of recombinant adeno-associated virus serotype 9 (AAV9) that expresses SAS (AAV9-SAS) into the bilateral ventricles of mouse brains. SAS enhanced the non-amyloidogenic processing of APP, thus reducing the levels of soluble Aβ and plaques in the 5xFAD mice. In addition, SAS significantly attenuated the cognitive deficits in 5xFAD mice, as demonstrated by novel object recognition and Morris water maze tests. Unlike other Aβ-cleaving proteases, SAS has highly strict substrate specificity. We propose that SAS can be an efficient modality to eliminate excessive Aβ from diseased brains.

## 1 Introduction

Alzheimer’s disease (AD) is a devastating neurodegenerative condition characterized by progressive memory loss and cognitive decline (Zhang et al., 2023). The distinctive pathological features of AD include the accumulation of amyloid plaques outside of neurons, and the presence of neurofibrillary tangles (NFTs) inside neurons, which are made of hyperphosphorylated tau (Zhang et al., 2023). The excessive accumulation of amyloid-β (Aβ) has been associated with these hallmarks in diseased brains, eventually leading to clinical symptoms (Selkoe and Hardy, 2016). Recent clinical advancements with Aβ immunotherapy strongly support this amyloid hypothesis (van Dyck et al., 2023). Consequently, viable strategies for treating AD might involve reducing the Aβ burden either by preventing its generation or by enhancing its clearance (Gandy and DeKosky, 2013).

Aβ is generated through the proteolytic cleavage of the amyloid precursor protein (APP) by β-secretase (BACE1) and γ-secretase (Selkoe and Hardy, 2016). Once produced via this amyloidogenic pathway, Aβ can adopt multiple conformational states, including monomers, oligomers, fibrils, and plaques. Oligomeric forms are considered the most toxic species among these various conformations (Cline et al., 2018). Oligomeric Aβ causes various cellular stresses, such as the inhibition of axonal transport, synaptic loss, and hyperphosphorylation of tau protein, leading to neuronal cell death (Cline et al., 2018; Fontana et al., 2020). Therefore, inhibitors of BACE1 and γ-secretase have been extensively tested in preclinical and clinical studies (Vassar, 2014). However, since BACE1 and γ-secretase also target several other endogenous substrates in addition to APP, their inhibition has resulted in severe off-target effects (Svedružić et al., 2013; Panza et al., 2019).

Under normal conditions, most APP is cleaved at the plasma membrane by α-secretase via a non-amyloidogenic pathway (Deng et al., 2013; Zhang et al., 2023), leading to the suppression of Aβ generation and the production of a secreted amino-terminal fragment of APP known as soluble APPα (sAPPα) (Kuhn et al., 2010). It has been demonstrated that the genetic upregulation of α-secretase prevents Aβ pathology by disrupting Aβ generation in several mouse models of AD. Additionally, small molecules that enhance α-secretase activity reduce Aβ accumulation in these mouse models (Etcheberrigaray et al., 2004; Chun et al., 2020). Furthermore, sAPPα has neurotrophic and neuroprotective properties (Habib et al., 2017; Mockett et al., 2017; Dar and Glazner, 2020). Reduced α-secretase activity has also been observed in the hippocampus of AD patients, as evidenced by decreased levels of sAPPα and C-terminal fragment-α (CTF-α) (Lichtenthaler, 2011; Marcello et al., 2012). Thus, boosting α-secretase activity has been considered a potential therapeutic strategy. As in the case of BACE1 and γ-secretase, α-secretases also target a considerable number of other endogenous proteins (Tucher et al., 2014). Consequently, unwanted off-target effects appear unavoidable with the elevation of α-secretase activity (Wetzel et al., 2017).

We previously reported that nuclear inclusion a (NIa) from turnip mosaic virus (TuMV) is a cytosolic protease with a strict substrate specificity for the sequence of Val-Xaa-His-Gln (Kang et al., 2001). A consensus substrate sequence, Val-His-His-Gln, is found at the α-cleavage site of Aβ. Inspired by this discovery, we have shown that NIa can act as an amyloid-β degrading enzyme by specifically cleaving both monomeric and oligomeric Aβ *in vitro* (Han et al., 2010; Shin et al., 2014). In the current study, we engineered a fusion protein where a heterologous signal peptide and a transmembrane domain were fused to the amino- and carboxy-termini of NIa, respectively. This fusion protein, which we refer to as synthetic α-secretase (SAS), cleaves APP at its α-site. It is known that several members of a disintegrin and metalloproteinase (ADAM) family, including ADAM9, ADAM10, and ADAM17, exhibit α-secretase activity (Lichtenthaler, 2011). These putative endogenous α-secretases exhibit broad substrate specificity, and their consensus substrate sequences are difficult to define (Tucher et al., 2014). In contrast, SAS may cleave only a limited number of substrates besides APP due to its highly strict substrate specificity. Stereotaxic injection of AAV9-SAS into the 5xFAD mouse model reduced Aβ burden and attenuated cognitive deficits. Overall, we found that SAS functions similarly to an α-secretase with potentially minimal off-target effects, and its activity can reduce Aβ load and improve cognitive function in an AD mouse model. Therefore, SAS represents a promising therapeutic approach to mitigate amyloid pathology.

## 2 Materials and methods

### 2.1 Plasmid construction

All DNA synthesis was performed at Integrated DNA Technologies (United States). Polymerase chain reaction (PCR) was performed utilizing nPfu-forte polymerase (Enzynomics, South Korea). APP_751_ and BACE1 cDNA were kind gifts from Dr. Doo Yeon Kim (MGH). All the following constructs were subcloned into pcDNA4mycHis. SAS is a fusion protein in which the catalytic domain (aa 56-445) of human BACE1 was replaced with the TuMV NIa proteolytic domain (aa 9-222). Sub(+) consists of amino-terminal portion of APP (aa 1-288), a substrate sequence (GYEVHHQSAA), and carboxy-terminal portion of BACE (aa 451-501). Sub(−) has an uncleavable substrate sequence (GYEVHAASAA). SASm has an Asp^128^-to-Asn mutation where the amino acid is a member of catalytic triad.

### 2.2 modRNA synthesis

Polyadenylated and capped modRNAs were synthesized by Trilink (United States) using 5-methyl-cytidine and pseudouridine, following phosphatase and DNase treatment. 5′ and 3′ UTRs designed by Trilink were used. Below is an open reading frame (ORF) sequence of DNA template.

Custom CleanCap™ SAS-HA modRNA:

ATGGCTCAGGCTCTCCCCTGGCTTTTGCTGTGGATGGGCGCGGGAGTTTTGCCTGCTCATGGTACTCAGCACGGTATTCGCCTTCCCCTTCGGAGTGGGTTGGGCGGCGCACCTCTGGGACTGCGCTTGCCTAGGGAGACAGACGAGGAGCCTGAAGAACCGGGTCGCGATTATAATCCGATTAGTAATAATATATGTCATCTCACGAACGTATCCGACGGTGCAAGCAACAGCCTGTACGGAGTCGGGTTTGGGCCGCTTATTTTGACAAACCGACATCTCTTTGAGAGAAATAATGGGGAGCTTGTCATCAAAAGTCGGCACGGAGAATTTGTAATAAAGAATACCACACAACTCCATCTTCTCCCGATTCCGGATCGCGATCTTCTCCTGATCAGGTTGCCGAAGGATATCCCTCCATTCCCGCAGAAACTTGGATTCAGGCAACCGGAAAAGGGAGAACGCATCTGCATGGTCGGGAGCAATTTCCAAACTAAGTCTATCACAAGCGTGGTGTCTGAAACTAGTACGATAATGCCAGTAGAGAATAGCCAGTTTTGGAAACATTGGATCAGCACGAAAGACGGACAATGCGGATCTCCGATGGTGTCAACTAAAGATGGTAAAATACTCGGCCTGCATTCACTTGCCAACTTCCAAAACTCAATTAACTATTTCGCCGCATTCCCTGACGACTTTGCCGAAAAGTACCTTCACACTATCGAGGCACATGAATGGGTAAAGCACTGGAAGTATAACACTAGCGCGATCTCATGGGGGTCACTGAATATTCAAGCGTCTCAGCCAAATATACCTCAAACGGACGAGAGCACTCTTATGACCATAGCTTATGTGATGGCTGCGATCTGTGCGCTCTTCATGCTTCCCTTGTGCCTCATGGTCTGTCAATGGCGATGTCTGAGGTGCCTTCGACAACAACATGATGATTTTGCGGACGATATTTCTCTTTTGAAATACCCATACGATGTTCCAGATTACGCTTGA.

### 2.3 AAV production

An AAV9 viral vector containing SAS gene under the control of the CMV promoter was generated by Virovek (United States), referred to as AAV9-SAS. For negative control, AAV9 containing GFP gene under the control of the CMV promoter (AAV9-GFP), and AAV9 virus-like particles (AAV9-VLP) were purchased from Virovek. The concentrations of the vectors were 2.12 × 10^13^ vg/mL (AAV9-SAS), 2.4 × 10^13^ vg/mL (AAV9-GFP), and 2.01 × 10^13^ vg/mL (AAV9-VLP).

### 2.4 Oligomeric FITC-Aβ42 preparation

Oligomeric FITC-Aβ_42_ solution was prepared by the method described previously (Shin et al., 2014). Briefly, 1 mg of FITC-Aβ_42_ peptides (4095738, Bachem, Switzerland) was dissolved in 1,1,1,3,3,3-hexafluoro-2-propanol (HFIP) (105228, Sigma-Aldrich, United States), followed by aliquoting into 10 microcentrifuge tubes. The solution was evaporated overnight in a fume hood to remove HFIP. The peptide film was stored at −80°C until used. Subsequently, the peptide was resolved in anhydrous dimethyl sulfoxide (DMSO) to a concentration of 1.25 mM. To enrich oligomers, the peptide solution was diluted in phenol-red free DMEM (LM001-59, Welgene, South Korea) to a final concentration of 100 μM, and the diluted solution was incubated at 4°C for 24 h. After incubation, the FITC-Aβ_42_ solution was treated to the SH-SY5Y cells as a concentration of 2.5 μM.

### 2.5 Transfection

DNA plasmids were transfected using Lipofectamine 2000 (Invitrogen, United States) for Ad293, and Lipofectamine LTX with Plus (Invitrogen, United States) for COS-7, SH-SY5Y in Opti-MEM (Gibco, United States) following the manufacturer’s instructions. The medium of Ad293, COS-7, and SH-SY5Y cells was changed to serum-free. Lipofectamine 2000 or Lipofectamine LTX with Plus and plasmid DNA were each diluted in Opti-MEM, and then mixed. The DNA transfectants were added to the cells. Medium was refreshed after 2 h for Ad293, but not for COS-7 and SH-SY5Y.

modRNA-SAS-HA was transfected by Lipofectamine RNAiMAX (Invitrogen, United States) in Opti-MEM. SH-SY5Y cells were grown in a 12-well culture plate until 80% confluency. Before transfection, the culture media were removed and SH-SY5Y cells were replenished with phenol-red free DMEM without serum. After 4 h, 3 μL of RNAiMAX reagent and 3.5 μg of modRNA were diluted into each 50 μL Opti-MEM. The two solutions were combined, and then incubated for 15 min at room temperature. The modRNA transfectants were added to the cells.

### 2.6 Cell culture and treatment

Ad293 cells were used for protein expression experiments. This cell line is a derivative of the commonly used HEK293 cell line with improved cell adherence and plaque formation properties. HEK293 cells are human embryonic kidney cells transformed by sheared adenovirus type 5 DNA. COS-7 cells were used for subcellular localization experiments. This cell line was obtained by immortalizing kidney cells of African green monkey with a modified SV40 virus. Human neuroblastoma, SH-SY5Y cells were used for the evaluation of SAS/SASm activity. This cell line is a thrice cloned subline of the neuroblastoma cell line SK-N-SH, which was established from a metastatic bone tumor from a 4-year-old cancer patient.

Ad293 and COS-7 cells were grown in Dulbecco’s Modified Essential Medium (DMEM) (Hyclone, United States) supplemented with 10% fetal bovine serum (FBS) (Hyclone, United States) and antibiotics/antimycotic. SH-SY5Y cells were cultured in DMEM with 10% FBS and 1% penicillin/streptomycin (Gibco, United States). The two cell lines were grown in a humidified incubator at 37°C with 5% CO_2_.

To check the expression of synthetic protein, Ad293 cells were transfected with NIa, BACE1, SAS, SASm plasmids using Lipofectamine 2000. The medium was refreshed after 2 h. After 24 h of transfection, the cells were harvested, and then analyzed by western blot. To characterize the subcellular localization of SAS, COS-7 cells were transfected with NIa, BACE1, SAS, SASm plasmids using Lipofectamine LTX with Plus. After 24 h of transfection, the cells were subjected to immunocytochemistry (ICC). For examination of SAS activity on APP, serum was depleted from SH-SY5Y cells. Subsequently, proteases and APP plasmids were co-transfected into SH-SY5Y cells using Lipofectamine LTX with Plus (Invitrogen, United States). After 24 h of transfection, the cells were harvested, followed by analysis by western blot. To assess enzymatic activity of SAS on oligomeric FITC-Aβ_42_, SH-SY5Y cells were transfected with SAS modRNA by Lipofectamine RNAiMAX (Invitrogen, United States). After 6 h, without changing the medium, oligomeric FITC-Aβ_42_ solution was added to the culture medium to a final concentration of 2.5 μM. After 12 h of oligomeric FITC-Aβ_42_ treatment, the cells were subjected to ICC.

### 2.7 Glycosidase treatment

Cell extracts (50 μg of protein) obtained from SAS expressing cells were denatured at 100°C for 10 min and treated with either PNGase F, Endopeptidase H, or O-glycosidase (New England Biolabs, United States) in GlycoBuffer (1X) at 37°C for 1 h. The samples were analyzed by western blot analysis.

### 2.8 Immunocytochemistry

The cells were fixed for 10 min with 1% paraformaldehyde followed by permeabilization with 0.15% Triton X-100 in PBS for 10 min. The cells were blocked with 3% BSA in PBS for 1 h, followed by incubation with primary antibodies (Supplementary Table S1) diluted in 3% BSA solution overnight at 4°C. Then, the cells were treated with fluorescent-labeled secondary antibodies (Supplementary Table S1) and Hoechst 33342 (H3570, Invitrogen, United States) in 3% BSA solution, followed by mounting with Faramount Aqueous Mounting Medium (S3205, Dako, United States). The slides were analyzed by confocal microscopy using FV3000 (Olympus, Japan). Z-stack images were acquired to examine the cleavage of FITC-Aβ_42_ peptides by SAS.

### 2.9 Animals

5xFAD transgenic mice for AD-associated amyloidogenesis, overexpressing human mutated APP (the Swedish mutation: K670N, M671L; the Florida mutation: I716V; the London mutation: V717I), and PS1 (M146L; L286V) (Oakley et al., 2006), were maintained on a mixed B6/SJL background. Animals were bred and kept in a temperature-controlled room at 20°C ± 2°C and a 12/12 h light–dark cycle (light on at 6 a.m.). Food and water were available *ad libitum*. To examine the effect of SAS on APP processing and Aβ deposition, AAV9-SAS was infused at 2 months of age by stereotaxic injection (*n* = 8 males) and AAV9-VLP was injected as a control (*n* = 8 males). The mice were sacrificed at 4 months of age and subjected to biochemical analysis and immunohistochemistry (IHC). Age-matched negative littermates were also used for western blot analysis. To assess the effect of SAS on cognitive deficits, AAV9-GFP, AAV9-SAS were infused into the brain of 5xFAD mice and the negative littermates at 4 months of age (WT/GFP: *n* = 2 males, *n* = 4 females; WT/SAS: *n* = 3 males, *n* = 4 females; 5xFAD/GFP: *n* = 1 males, *n* = 5 females; 5xFAD/SAS: *n* = 1 male, *n* = 7 females). The mice were transferred to the animal facility at Chonnam National University (CNU) at 6 months of age. Behavioral tests were performed at Dr. Won Suk Choi’s laboratory to analyze the memory function of the mice from 6.5 months of age. All animal experiments were approved by the ethics committee at Gwangju Institute of Science and Technology (GIST), and performed in compliance with the institutional guidelines.

### 2.10 Stereotaxic injection

Mice were anesthetized by intraperitoneal injection of a mixture of 95 mg/kg ketamine 50 (Yuhan, Korea) and 5 mg/kg xylazine (Bayer, Germany), and positioned in a stereotaxic frame (RWD Life Science, United States). Lidocaine was applied subcutaneously before exposure of the skull. Small holes were drilled onto the skull at the coordinates of the injection sites. A 10 μL of viral vectors were infused into bilateral ventricles (AP, −0.46; ML, ±1.00; DV, −2.20 from Bregma in mm) at a flow rate of 2 μL/min using UltraMicroPump III (World Precision Instruments, United States). After injection, the needle was left in place for 15 min, and then slowly withdrawn.

### 2.11 Tissue preparation

For biochemical/histological analysis, the mice injected with AAV9 vectors were anesthetized by avertin (250 mg/kg, i.p. injection), and transcardially perfused with PBS, followed by extraction of brain. The brain was dissected into each hemisphere through midsagittal plane. The hippocampus of left hemisphere was segregated for biochemical analysis, and snap-frozen in liquid nitrogen, and stored at −80°C until following experiments. Subsequently, the hippocampus was homogenized using a homogenizer in RIPA buffer containing a proteases inhibitor cocktail (535140, Sigma, United States), followed by sonication for 4 s, and centrifugation for 10 min at 4°C at 15,000 × g. The supernatant was used for western blot analysis. For enzyme linked immunosorbent assay (ELISA) analysis, the supernatant was ultracentrifuged for 1 h at 4°C at 100,000 × g. The final supernatant was used for the ELISA assay. The samples were stored at −80°C until use. Right hemisphere was post-fixed with 4% paraformaldehyde (PFA) at 4°C for 24 h, followed by washing with PBS at 4°C for 24 h. The hemisphere was cryoprotected in 30% sucrose (in PBS, pH 7.4) at 4°C for 48–72 h, followed by embedding in OCT compound (3801480, Leica, Germany). The frozen brain was sectioned into 40 μm thick slices with a cryostat microtome (HM525NX, Thermo Scientific, United States). The sections were collected in a cryoprotective solution (23.5% glycerol, 28.5% ethylene glycol in PBS), and stored at −20°C until used.

### 2.12 Immunohistochemistry

The free-floating sections were incubated in 5% goat serum (ab7481, Abcam, United States), 5% BSA, 0.15% Triton X-100 in PBS for 1 h with agitation at room temperature for blocking and permeabilization. Then, the sections were subjected to antigen retrieval in citrate buffer (10 mM trisodium citrate, 0.05% tween 20, pH 6.0) using a steamer (IW-1102A, IHC world, United States) for 40 min. The sections were reacted with primary antibodies (Supplementary Table S1) at 4°C with agitation for 48 h. After incubation, the sections were washed with PBS and reacted with fluorescent-labeled secondary antibodies (Supplementary Table S1) and Hoechst 33342 dye for 1 h. The sections were washed with PBS and mounted with ProLong Glass Antifade Mountant (P36980, Invitrogen, United States). The slides were imaged by confocal microscopy for representative images. For quantification of Aβ plaques, the sagittal sections containing hippocampus regions were scanned by a research slide scanner (VS200, Olympus, United States). Then, the area covered by MOAB-2 fluorescence signals (A_MOAB-2_, μm^2^) from the hippocampus and total area of the hippocampus (A_total_, μm^2^) were measured by ImageJ software (NIH). The MOAB-2 area was calculated by the below equation.

$$\mathrm{MOAB}-2\;\mathrm{area}\;\left(\%\right)=\frac{A_{\mathrm{MOAB}-2}}{A_{\mathrm{total}}}\times 100$$

The average greyscale values of all pixels from the hippocampal region were measured using ImageJ and represented as mean MOAB-2 intensity. The total number of plaques (*N*_total_) were measured utilizing the Analyze Particle function of ImageJ software. Plaque number was calculated by following equation.

$$\mathrm{Plaque}\;\mathrm{number}\;\left(\mathrm{plaques}/{\mathrm{mm}}^{2}\right)=\left(\frac{N_{\mathrm{total}}}{A_{\mathrm{total}}}\right)\times 1000000$$

Three sections were used to calculate average values of each animal, and four to five mice were used for calculation of mean MOAB-2 area and plaque number and for statistical analysis.

### 2.13 Protein electrophoretic analysis and western blotting

For the samples from cells, the media and cells were harvested after 24 h of transfection. Media were collected, diluted with SDS sample buffer, and (20 μg of protein) were separated on SDS-PAGE using 10% acrylamide gel. The cells were harvested and lysed by sonication in RIPA buffer (150 mM sodium chloride, 1% Triton X-100, 0.5% sodium deoxycholate, 0.1% SDS, 50 mM Tris, pH 8.0) containing a proteases inhibitor cocktail. Then, the cells were centrifuged for 10 min at 4°C at 15,000 × g. Supernatants (20 μg of protein) were separated on SDS-PAGE using 10% acrylamide gel. Tissue lysate (20 μg of protein) were separated on SDS-PAGE using Pepti-Gel Peptide PAGE Analysis Kit (EBA-1053, ELPIS Biotech, South Korea) for analysis of APP CTF-β or 10% acrylamide gel for analysis of other proteins. The proteins were transferred to a PVDF membrane (IPVH00010, Millipore, United States) for immunoblotting. After blocking with 5% Skim milk in TBS-T, the membranes were incubated with primary antibodies (Supplementary Table S1) overnight at 4°C. The membranes were further incubated with secondary antibodies conjugated with HRP (Supplementary Table S1) for 1 h at room temperature, and developed using EZ-Western Lumi-Pico Kit (DG-WP250, DoGenBio, South Korea) or Western Femto ECL Kit (FEMTO-100, LPS solution, South Korea). Signals were detected by Amersham^™^ ImageQuant^™^ 800 (IQ800, Cytiva, Germany) and analyzed and quantified using ImageQuant^™^ TL software (Cytiva, Germany).

### 2.14 Enzyme linked immunosorbent assay

In a blinded fashion, concentration of Aβ_40_ and Aβ_42_ was measured by ME using Human Amyloid-β (1-40) (FL) Assay Kit (27718, Immuno-Biological Laboratories, Japan) and Human Amyloid-β (1-42) (FL) Assay Kit (27719, Immuno-Biological Laboratories, Japan), respectively. The procedures were performed according to the respective supplier instructions. The optical density of samples was measured by SpectraMax^®^ ABS Plus (Molecular Devices, United States).

### 2.15 Behavior test

The mice injected with AAV9 vectors at 4 months of age were subjected to behavior tests at the age of 6.5 months of age. All behavioral experiments were performed by B-RM from W-SC’s laboratory in a blinded manner.

#### 2.15.1 Open field test

To measure locomotor activity, the open field test (OFT) was performed in an open-top opaque box (36 × 36 × 40 cm). Mice were placed at the center of the arena, and were habituated in the arena for 5 min. The next day, the mice were allowed to freely explore the arena for 20 min under recording. The video was analyzed to measure the locomotion activity using ANY-maze software (Stoelting, United States).

#### 2.15.2 Novel object recognition test

The novel object recognition (NOR) test was performed in an open-top opaque box (36 × 36 × 60 cm). During the training phase, two identical objects were placed at the center of the box with a distance between them. Mice were placed between the objects and were explored for 8 min. During the test phase at the next day, one object was replaced with a novel object, and the other object which had used in the training phase remained. Mice were placed between the objects again and explored for 8 min while being recording. The video was analyzed to measure the exploring time of the familiar and novel objects using ANY-maze software. The preference index for the novel object was calculated as the exploring time spent on the novel object (n) divided by the total exploring time for both novel and familiar objects (n + f)

$$\mathrm{Preference}\;\mathrm{index}\;\left(\%\right)=\frac{n}{n+f}\times 100$$

#### 2.15.3 Morris water maze test

Morris water maze (MWM) test was performed using a circular water pool (120 cm in diameter) filled with opaque water. Two visual cues were attached to the wall of the pool. The escape platform was placed in one quadrant 1.5 cm below the surface of water. For the acquisition phase, mice were subjected to 4 trials per day for 4 consecutive days. In each trial, mice were placed in any of the quadrants of the pool except the quadrant with the platform, and then allowed to swim for 60 s under recording. Mice that did not reach the platform within 60 s were shifted to the platform and allowed to stay for 10 s to remember the location. For the reversal trials, mice were subjected to 4 trials per day for 2 consecutive days starting 3 days after the acquisition phase. The platform was placed in the opposite quadrant. Mice were allowed to swim and find the shifted escape platform for 60 s under recording. The video was analyzed to track the mice and measure the latency to reach the platform and time spent in each quadrant by ANY-maze.

### 2.16 Statistics

All the data are presented as the mean ± standard error of the mean (SEM). Statistical analysis was performed using OriginPro 2022 (OriginLab Corporation, United States) or Prism 9 (GraphPad Software, United States). Using Grubbs’ test, outliers were detected and removed from the dataset. Levene’s test was performed to determine the homogeneity of the variances between the groups that are statistically compared. Shapiro–Wilk test was employed to perform a normality test. To compare WT, 5xFAD/VLP, and 5xFAD/SAS in western blot results, data were analyzed by one-way analysis of variance (ANOVA) or Brown-Forsythe ANOVA followed by Bonferroni’s or Dunnett’s T3 multiple comparisons test. To analyze results from behavioral tests, data were analyzed by one-way ANOVA followed by Tukey multiple comparisons test. For all analysis, statistical significance was set at a *p*-value <0.05.

## 3 Results

### 3.1 Conceptualization and characterization of synthetic α-secretase

**Figure 1:**
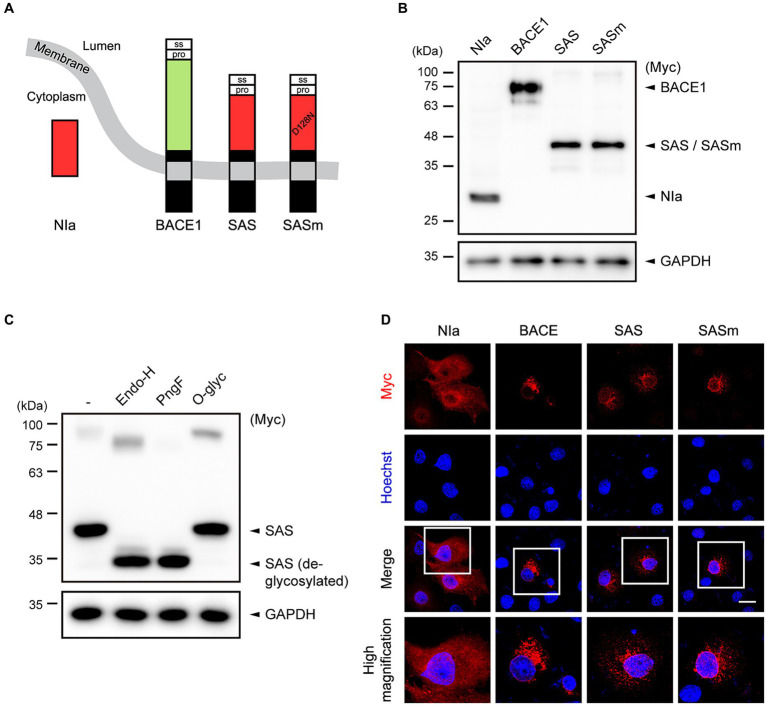
The design and characterization of SAS. **(A)** Schematic illustration of the concept of SAS. The signal sequence (ss) and prodomain (pro) derived from human BACE1 were fused to the N-terminus of NIa. In addition, the transmembrane and intracellular domains derived from BACE1 were fused to the C-terminus of NIa. SASm is a catalytically inactive mutant (D128N) form of SAS. **(B)** Cell lysates from Ad293 cells transfected with plasmids encoding NIa, BACE1, SAS, or SASm were subjected to western blotting. GAPDH was used as a loading control. **(C)** Cell lysates from Ad293 cells transfected with plasmids encoding SAS were treated with two N-glycosidases, Endoglycosidase H (Endo-H) and PNGase F (PngF), or an O-glycosidase (O-glyc), and subjected to western blotting. GAPDH was used as a loading control. **(D)** COS-7 cells transfected with NIa, BACE1, SAS, or SASm plasmids were monitored by an anti-Myc antibody and an anti-mouse IgG antibody conjugated with Alexa555 (red) to investigate their subcellular localization. Scale bar, 20 μm.

NIa is a cytosolic protease that can cleave Aβ in a highly specific manner (Han et al., 2010; Shin et al., 2014). In the current study, we sought to express NIa in intracellular vesicles including the endoplasmic reticulum (ER), Golgi apparatus, and endosomes where Aβ is generated from APP or extracellular Aβ is endocytosed. When modified to be expressed in these intracellular vesicles, NIa would prevent the generation of Aβ from APP or enhance the degradation of endocytosed Aβ. A cytosolic protein can often be driven to the secretory pathway by utilizing a heterologous signal peptide. Therefore, we fused the signal sequence derived from human BACE1 to the N-terminus of NIa. In addition, we fused the transmembrane and intracellular domains derived from BACE1 to the C-terminus of NIa (Figure 1A). We hypothesized that the resulting fusion protein might function like an α-secretase by cleaving APP at the α-site, thus being referred to as synthetic α-secretase (SAS). The intracellular domain of BACE1 contains signals that are required to guide BACE1 through the secretory pathway and are involved in retention to endosome (Cole and Vassar, 2007). Since SAS possesses this intracellular domain, it may co-localize and compete with BACE1 in various vesicular compartments for processing APP. As a negative control, we also generated a mutant form of SAS, SASm, in which the catalytic triad is defective due to a Asp^128^-to-Asn substitution (Figure 1A).

To evaluate the expression of SAS, Ad293 cells were transfected with plasmids expressing Myc-tagged NIa, BACE1, SAS, and SASm, and then the lysates were analyzed by western blotting. NIa and BACE1 were detected by anti-Myc antibody, and their apparent molecular sizes were 27 kDa and 75 kDa, respectively, as expected. SAS and SASm were also detected by anti-Myc antibody as a single band, but their apparent molecular sizes, 45 kDa, were larger than the calculated sizes, 35.5 kDa (Figure 1B). This discrepancy between the observed and calculated molecular sizes might be due to post-translational modification of SAS and SASm. Web-based algorithms (Gupta and Brunak, 2001; Steentoft et al., 2013) predicted three N-glycosylation sites (N^70^, N^115^, and N^253^) and two O-glycosylation sites (S^32^ and T^47^) in SAS. To test whether SAS is glycosylated, lysates of Ad293 cells expressing SAS were treated with two N-glycosidases, endoglycosidase-H (Endo-H) and PNGase-F (PngF), and one O-glycosidase (O-glyc). The apparent molecular size of SAS was reduced closely to the calculated size upon the treatment with N-glycosidases but not with O-glycosidase, which implies that SAS is N-glycosylated when expressed in mammalian cells (Figure 1C). The faint upper bands likely represent the dimeric form of SAS.

To assess the intracellular localization of SAS, COS-7 cells were transfected with plasmids expressing Myc-tagged NIa, BACE1, SAS, and SASm, and then were analyzed by immunocytochemistry (ICC). As expected, NIa was localized in the cytosol, and BACE1 in ER/Golgi-like structures and endosome-like puncta. SAS and SASm were mainly localized in vesicular structures as BACE1 was, but not in the cytosol (Figure 1D). Overall, these data indicate that SAS and SASm were subjected to N-glycosylation and localized in intracellular vesicles such as the ER, Golgi, and endosomes like BACE1.

### 3.2 SAS cleaves an artificial substrate and APP at the α-site

**Figure 2:**
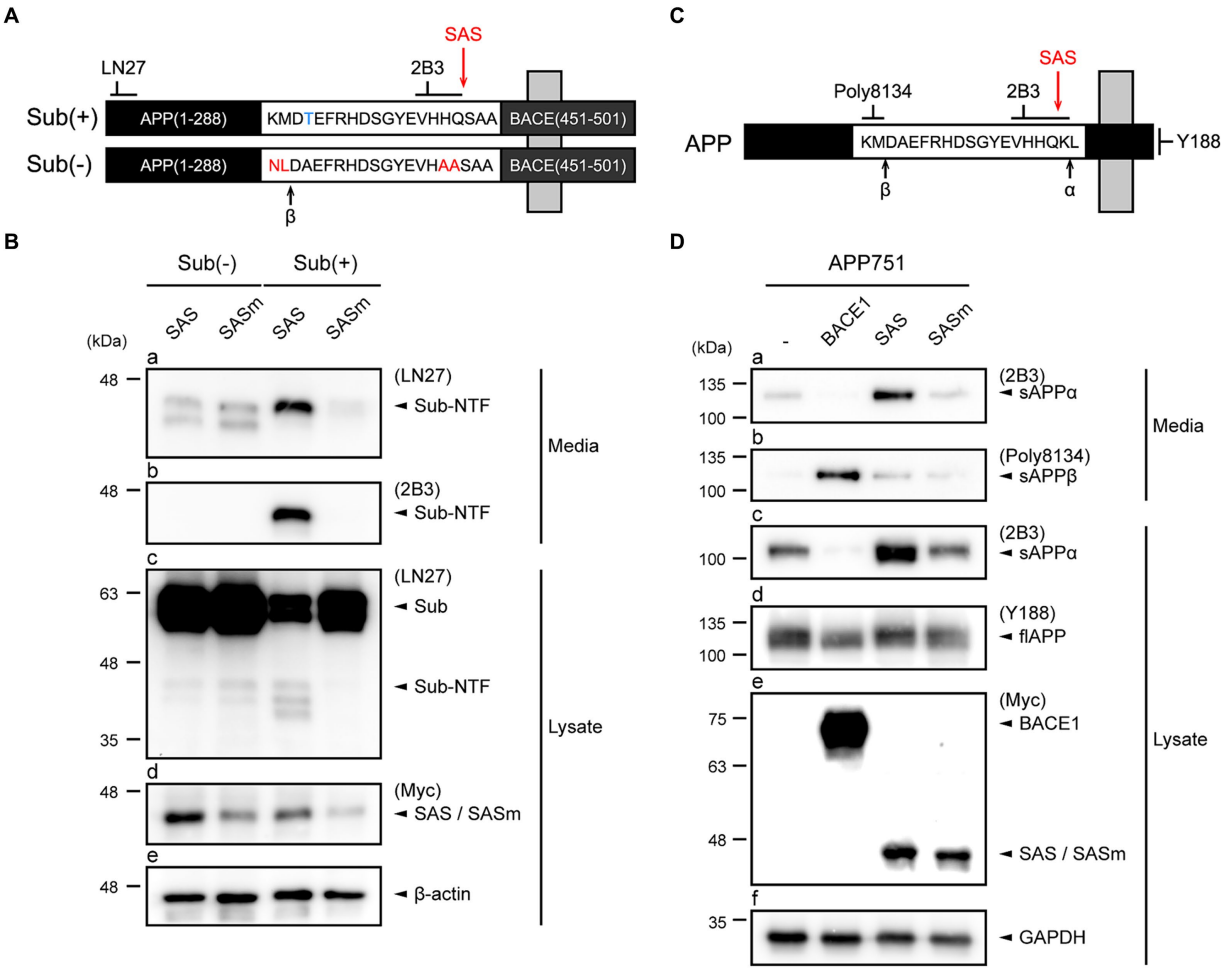
SAS cleaves an artificial type-I membrane protein and APP at the α-site. **(A)** Schematic representation of an artificial membrane protein substrate designed to monitor SAS activity. The Sub(+) protein contains a substrate sequence (VHHQ/S) that can be cleaved by SAS, whereas Sub(−) protein contains a non-cleavable sequence (VHAAS). These substrate sequences are flanked by the N-terminal fragment of APP and the C-terminal fragment of BACE1. Cleavage sites of β-secretases, and SAS are indicated with arrows. **(B)** SH-SY5Y cells were co-transfected with substrates, Sub(−) or Sub(+), and proteases, SAS or SASm, and then analyzed by western blotting. LN27 detects full-length substrates (Sub) and the N-terminal fragment of Sub(+) (Sub-NTF). 2B3 detects the C-terminus of sAPPα. The expression of SAS and SASm were detected by an anti-Myc antibody. GAPDH was used as a loading control. **(C)** Schematic representation of APP. Epitopes of antibodies used to monitor APP processing are shown. Cleavage sites of α-, β-secretases, and SAS are indicated with arrows. **(D)** Cell lysates and culture media were prepared from SH-SY5Y cells co-transfected with plasmids encoding APP and proteases (BACE1, SAS, or SASm). On western blots, sAPPα, sAPPβ, and full-length APP were detected with antibodies 2B3, poly8134, and Y188, respectively. The expressions of proteases were detected by an anti-Myc antibody. GAPDH was used as a loading control.

To evaluate the α-secretase-like activity of SAS, we first generated an artificial type-I membrane protein substrate, Sub(+), that structurally resembles APP. Sub(+) harbors a short stretch of amino acids containing the optimal substrate sequence (VHHQ/S; “/” indicates the scissile bond) of NIa that was flanked by the N-terminus of APP (aa 1-288) and the transmembrane and intracellular domains of BACE1 (aa 451-501) (Figure 2A). A negative control substrate, Sub(−), harbors a sequence (VHAAS) that cannot be cleaved by NIa. In addition, the β-cleavage site is removed in Sub(+), while it is enhanced by incorporating a Swedish mutation (NL/DA) in Sub(−). Two antibodies were utilized to detect the cleavage of the artificial substrates on western blotting: LN27 was specific to the N-terminus of APP, and 2B3 to the C-terminus of sAPPα (Figure 2A).

SH-SY5Y cells were transfected with plasmids expressing Sub(+) or Sub(−) and plasmids expressing Myc-tagged SAS or SASm. The cells and culture media were separately collected, and then subjected to western blotting. The western blots probed by LN27 revealed that a protein band with a size smaller than that of full-length Sub(+) was present in media only when Sub(+) and SAS were co-expressed (Figure 2B, panel a). The same protein band was detected by 2B3, implying that it was produced by the cleavage of Sub(+) at the α-site by SAS (Figure 2B, panel b). Two faint bands were detected when Sub(−) was co-expressed with either SAS or SASm (Figure 2B, panel a). These bands may be produced by the cleavage of Sub(−) by endogenous β-secretases. The full-length Sub(+) or Sub(−) substrates were detected in lysates by LN27. No cleavage in Sub(−) was detected in lysates when Sub(−) was co-expressed either with SAS or SASm (Figure 2B, panel c, lanes 1–2). The level of the full-length Sub(+) was significantly reduced when it was co-expressed with SAS but not with SASm (Figure 2B, panel c, lanes 3–4). These data indicate that SAS cleaves an artificial membrane protein substrate at the α-site.

To study the effect of SAS on APP, SH-SY5Y cells were co-transfected with plasmids expressing full-length APP_751_ and also plasmids expressing Myc-tagged BACE1, SAS, or SASm. We utilized 2B3 and Poly8134 to detect the cleavage products of APP, sAPPα and the soluble APPβ (sAPPβ), respectively. The antibody specific to the C-terminus of APP, Y188, was also utilized to detect the full-length APP (Figure 2C). Co-expression of APP_751_ with SAS, but not with SASm, resulted in the increased levels of sAPPα in media (Figure 2D, panel a) and lysates (Figure 2D, panel c). When APP_751_ and BACE1 were co-expressed, sAPPβ, but not sAPPα, was generated (Figure 2D, panel b). The expressions of full-length APP, BACE1, SAS, and SASm were confirmed (Figure 2D, panels d,e). Taken together, these results indicate that SAS can specifically cleave APP at the α-site.

### 3.3 SAS cleaves FITC-Aβ_42_ at the α-site

**Figure 3:**
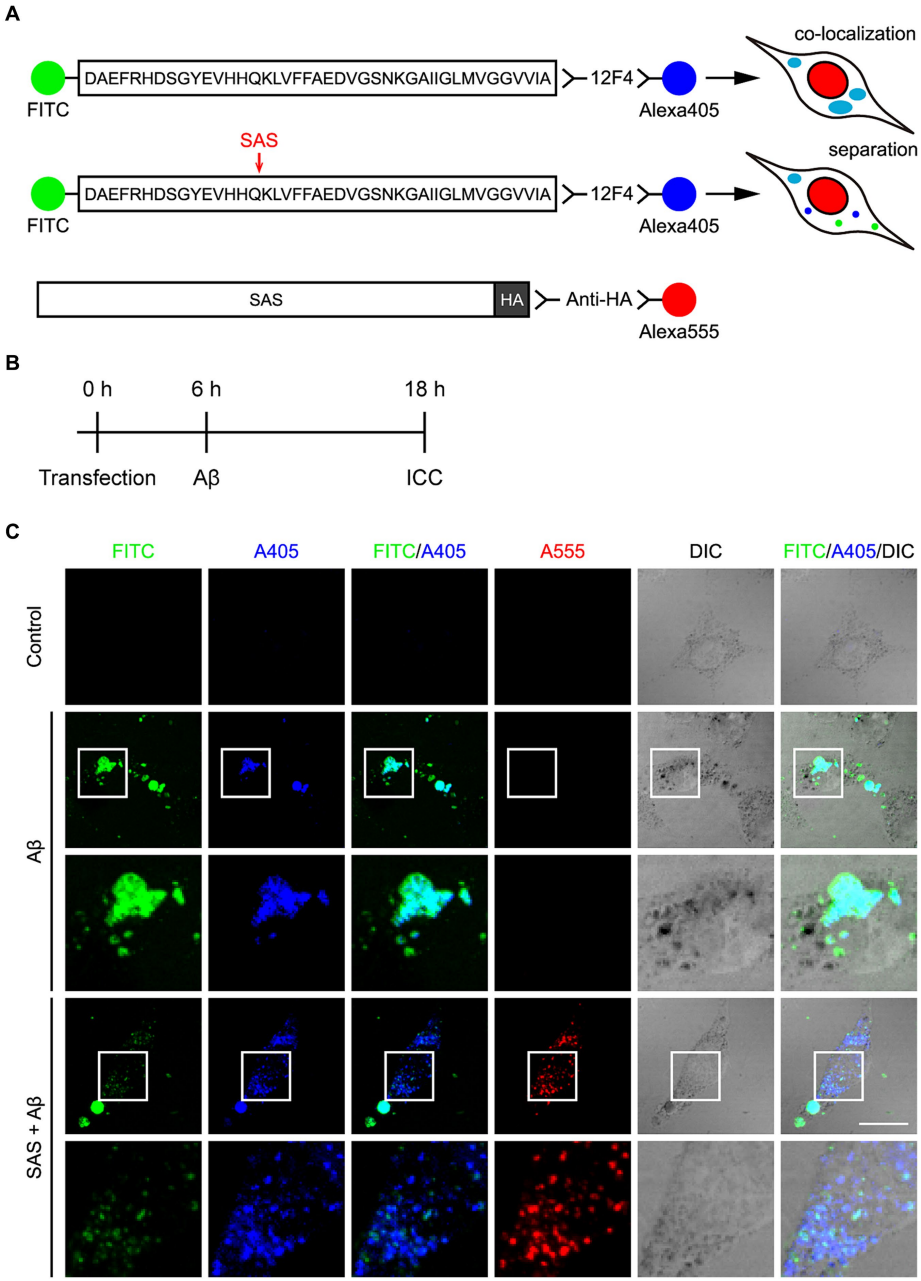
SAS cleaves FITC-Aβ_42_ at the α-site. **(A)** Schematic diagram of the experimental design and hypothesis to examine the activity of SAS on Aβ peptides. The N-terminus of Aβ_42_ peptides was detected by labeled FITC and the C-terminus was detected by 12F4 and a secondary antibody conjugated with Alexa405. Intact Aβ would be detected without SAS expression by the co-localization of FITC and Alexa405 with a cyan color in ICC. Cleavage of Aβ would be detected with SAS expression by separated signals of FTIC and Alexa405. SAS expression was monitored by an anti-HA antibody and a secondary antibody conjugated with Alexa555. **(B)** Experimental timeline for transfection, treatment with Aβ peptides, and immunocytochemistry (ICC). **(C)** After transfection and Aβ treatment, SH-SY5Y cells were subjected to ICC with 12F4 (blue) and anti-HA antibody (red), followed by Z-stack acquisition with maximum intensity projection. The images in the white lined open boxes are represented at a higher magnification in the adjacent rows. Scale bar, 20 μm. DIC, differential interference contrast.

As another approach to evaluate the activity of SAS, we utilized an Aβ_42_ peptide labelled with FITC at its N-terminus (FITC-Aβ_42_). The C-terminus of FITC-Aβ_42_ was detected by 12F4 and a secondary anti-mouse IgG antibody labelled with Alexa405. Thus, the N- and C-termini of Aβ_42_ can be detected under a fluorescent microscope by FITC and Alexa405, respectively. The treated FITC-Aβ_42_ was efficiently uptaken by SH-SY5Y cells. We presumed that the intact FITC-Aβ_42_ is monitored by the co-localization of FITC and Alexa405, while a cleavage in FITC-Aβ_42_ is detected by the loss of co-localization of the two fluorescent dyes (Figure 3A).

SH-SY5Y cells were transfected with modRNA expressing HA-tagged SAS (SAS modRNA), whose expression was monitored by an anti-HA antibody and a secondary anti-rat IgG antibody labelled with Alexa555 (Figure 3A). At 6 h post-transfection with the SAS modRNA, the cells were treated with 2.5 μM oligomeric FITC-Aβ_42_ for 12 h, and then were observed under a fluorescent microscope (Figure 3B). Without SAS, FITC and Alexa405 were co-localized in swollen vesicles (Figure 3C, rows 2–3; Supplementary Figures S1A,B) implying that the treated FITC-Aβ_42_ remained intact in cells. The morphological changes of vesicles are also consistent with previous reports that Aβ_42_ induces endosomal and lysosomal swelling in neuronal cells (Riera-Tur et al., 2022). On the contrary, upon the treatment with SAS modRNA, the co-localization of FITC and Alexa405 was significantly reduced with most of the two fluorescences being separated in small-sized puncta (Figure 3C, rows 4–5; Supplementary Figures S1A,B). It was of note that the vesicular swelling was also significantly inhibited by SAS modRNA. Areas containing approximately 1,000 cells were further scanned at lower magnification. Upon the transfection with the SAS modRNA, the co-localization of FITC and Alexa405 was reduced by about 50% (Supplementary Figure S1C). These data indicate that SAS cleaves Aβ_42_ peptides at the α-site in vesicles.

### 3.4 Expression of SAS in the 5xFAD mice injected with AAV9-SAS

**Figure 4:**
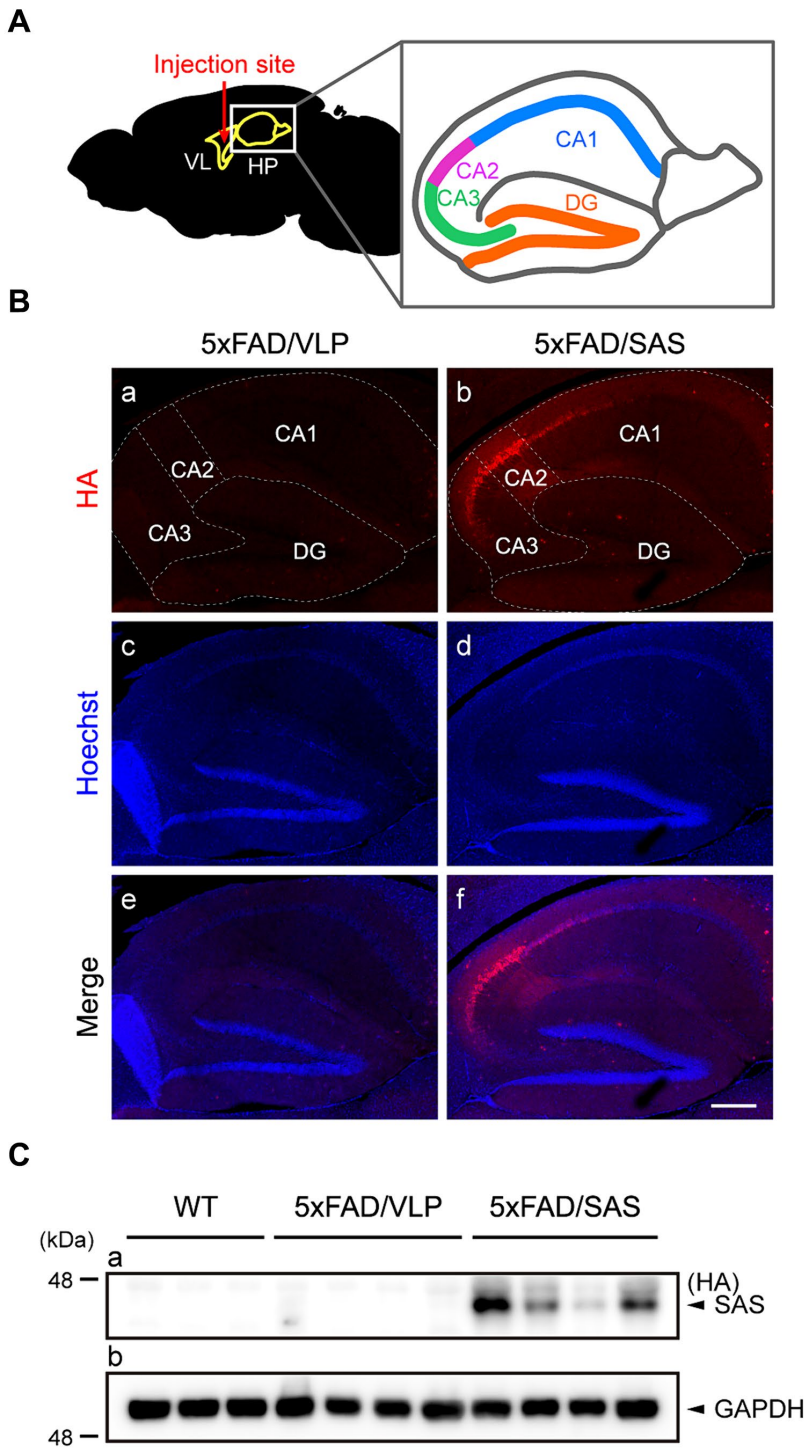
Expression of SAS in the 5xFAD mice injected with AAV9-SAS. **(A)** Schematic illustration of the injection site for stereotaxic injection and a detailed description of hippocampus anatomy. VL, lateral ventricle; HP, hippocampus; CA, cornu ammonis; DG, dentate gyrus. **(B)** Immunohistochemistry (IHC) was performed with an anti-HA antibody (red) to examine the expression of SAS in the 5xFAD mice. Representative images are shown. Scale bar, 300 μm. **(C)** Hippocampal lysates from the 5xFAD and age-matched WT mice were subjected to western blotting and detected by an anti-HA antibody. SAS was expressed in AAV9-SAS injected mice. GAPDH was used as a loading control. Male mice were used (WT, *n* = 3; 5xFAD/VLP, *n* = 4; 5xFAD/SAS, *n* = 4).

To evaluate the therapeutic potential of SAS in the 5xFAD mice, we performed stereotaxic injection of recombinant AAV vectors into the bilateral ventricles adjacent to the hippocampus (Figure 4A) that is associated with major cognitive functions. The recombinant AAV9 vector expressing SAS with HA-tag under the control of the CMV promoter, AAV9-SAS, was utilized for this experiment, and an empty capsid of AAV9, AAV9-VLP, was used as a control. The vectors were delivered at 2 months of age, and the mice were then sacrificed and analyzed at 4 months of age.

Immunohistochemistry (IHC) using an anti-HA antibody revealed that SAS was expressed in the hippocampus, especially intensively in the CA2/3 region (Figure 4B, panels b,f). More specifically, the expression of SAS was observed in the stratum oriens, the stratum radiatum, and the stratum lacunosum moleculare, whereas there was little expression of SAS in the stratum lucidum (Supplementary Figure S2). Western blotting of the hippocampal extracts using an anti-HA antibody also confirmed the expression of SAS in the hippocampus (Figure 4C). Altogether, SAS was successfully expressed in the hippocampus, specifically in the CA2/3 region, after the stereotaxic injection of AAV9-SAS.

### 3.5 SAS enhances the non-amyloidogenic processing of APP in the 5xFAD mice

**Figure 5:**
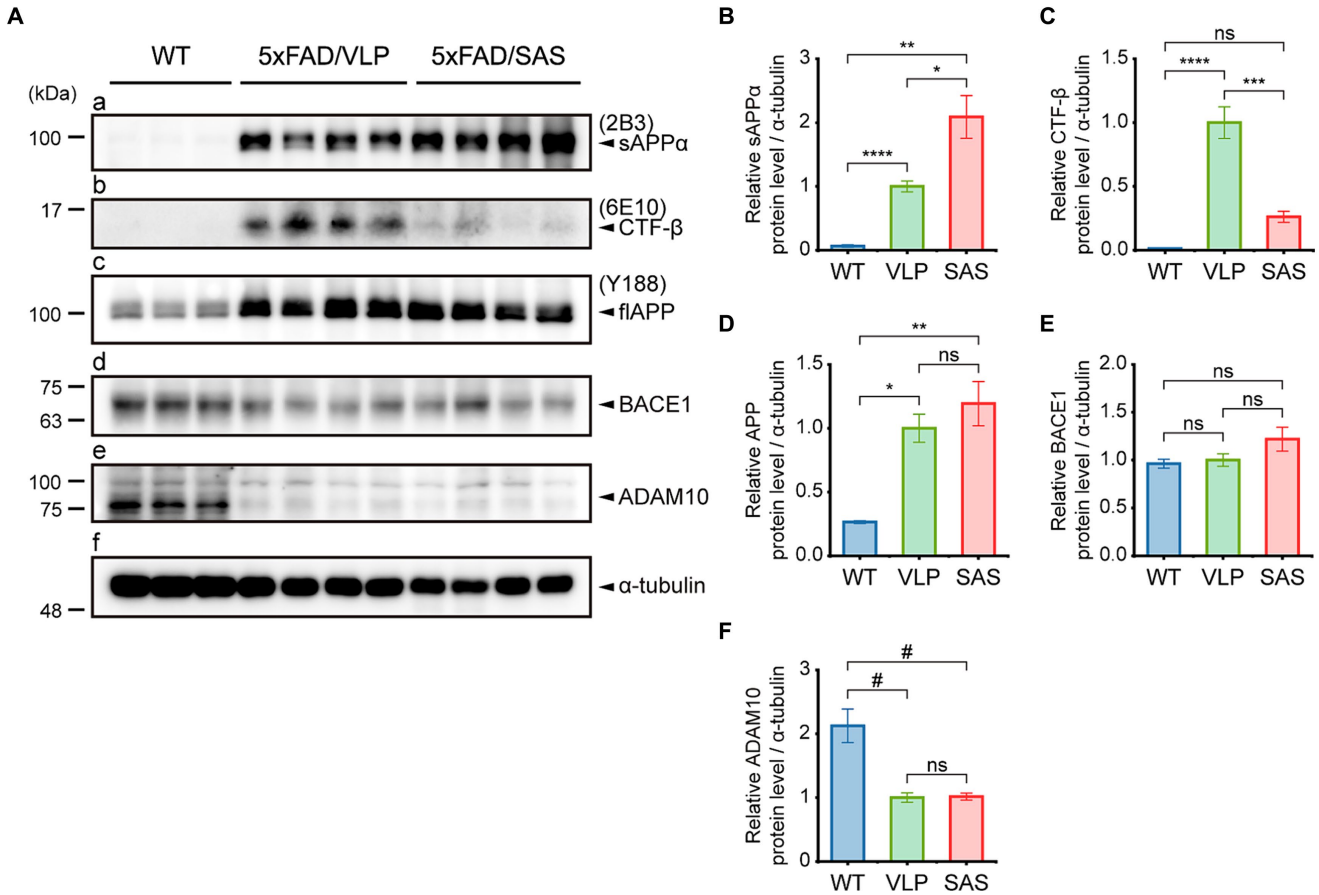
SAS enhances the non-amyloidogenic processing of APP in the 5xFAD mice. **(A)** Hippocampal extracts from the 5xFAD and age-matched WT mice were analyzed by western blotting. Representative blots are shown. Products of APP processing (sAPPα and CTF-β) and full-length APP were detected by the 2B3, 6E10, and Y188 antibodies, respectively. Endogenous proteases (BACE1 and ADAM10) associated with APP processing were detected by an anti-BACE1 antibody and an anti-ADAM10 antibody, respectively. α-tubulin was used as a loading control. **(B–F)** Western blotting results were quantified and statistically analyzed (normalized by the amount of α-tubulin). **(B)** Densities of sAPPα (Brown-Forsythe ANOVA followed by Dunnett’s T3 post-hoc test: WT vs. 5xFAD/VLP, *p* < 0.0001; WT vs. 5xFAD/SAS, *p* = 0.0015; 5xFAD/VLP vs. 5xFAD/SAS, *p* = 0.0376), **(C)** CTF-β (one-way ANOVA followed by Bonferroni’s post-hoc test: WT vs. 5xFAD/VLP, *p* < 0.0001; WT vs. 5xFAD/SAS, *p* = 0.5124; 5xFAD/VLP vs. 5xFAD/SAS, *p* = 0.0001), **(D)** full-length APP (Brown-Forsythe ANOVA followed by Dunnett’s T3 post-hoc test: WT vs. 5xFAD/VLP, *p* = 0.0008; WT vs. 5xFAD/SAS, *p* = 0.0067; 5xFAD/VLP vs. 5xFAD/SAS, *p* = 0.7558), **(E)** BACE1 (one-way ANOVA: ns, no significance), and **(F)** ADAM10 (one-way ANOVA followed by Bonferroni’s post-hoc test: WT vs. 5xFAD/VLP, *p* < 0.0001; WT vs. 5xFAD/SAS, *p* < 0.0001; 5xFAD/VLP vs. 5xFAD/SAS, *p* > 0.9999) were analyzed. WT, wild type; VLP, 5xFAD/VLP; SAS, 5xFAD/SAS. ^*^p < 0.05, ^**^p < 0.01, ^***^p < 0.005, ^****^p < 0.001, ^#^*p* < 0.0005, and ^##^*p* < 0.0001. Each bar and error bar represents the mean ± standard error of the mean (SEM). Male mice were used (WT, *n* = 3; 5xFAD/VLP, *n* = 8; 5xFAD/SAS, *n* = 8).

We examined whether SAS affected the amyloidogenic versus non-amyloidogenic processing of APP in the 5xFAD mice by western blotting (Figure 5). The sAPPα level was elevated approximately by 2-fold by AAV9-SAS as detected with 2B3, indicating that SAS enhanced the cleavage of APP at the α-site (Figure 5A, panel a; Figure 5B). The C-terminal fragment of APP cleaved at the β-site, CTF-β, was detected with 6E10. The CTF-β level was profoundly reduced by AAV9-SAS (Figure 5A, panel b; Figure 5C). Since the BACE1 level was unaltered (Figure 5A, panel d; Figure 5E), these data suggested that either less CTF-β was produced or the produced CTF-β was further cleaved at the α-site in the presence of SAS. In either case, the results confirmed that SAS enhanced the cleavage of APP or its fragments at the α-site. The expression levels of full-length APP, BACE1, and an α-secretase (ADAM10) were not significantly affected by SAS (Figure 5A, panels c–e; Figures 5D–F). Overall, the AAV-mediated expression of SAS enhanced the non-amyloidogenic pathway of APP processing.

### 3.6 SAS reduces Aβ load in the 5xFAD mice

**Figure 6:**
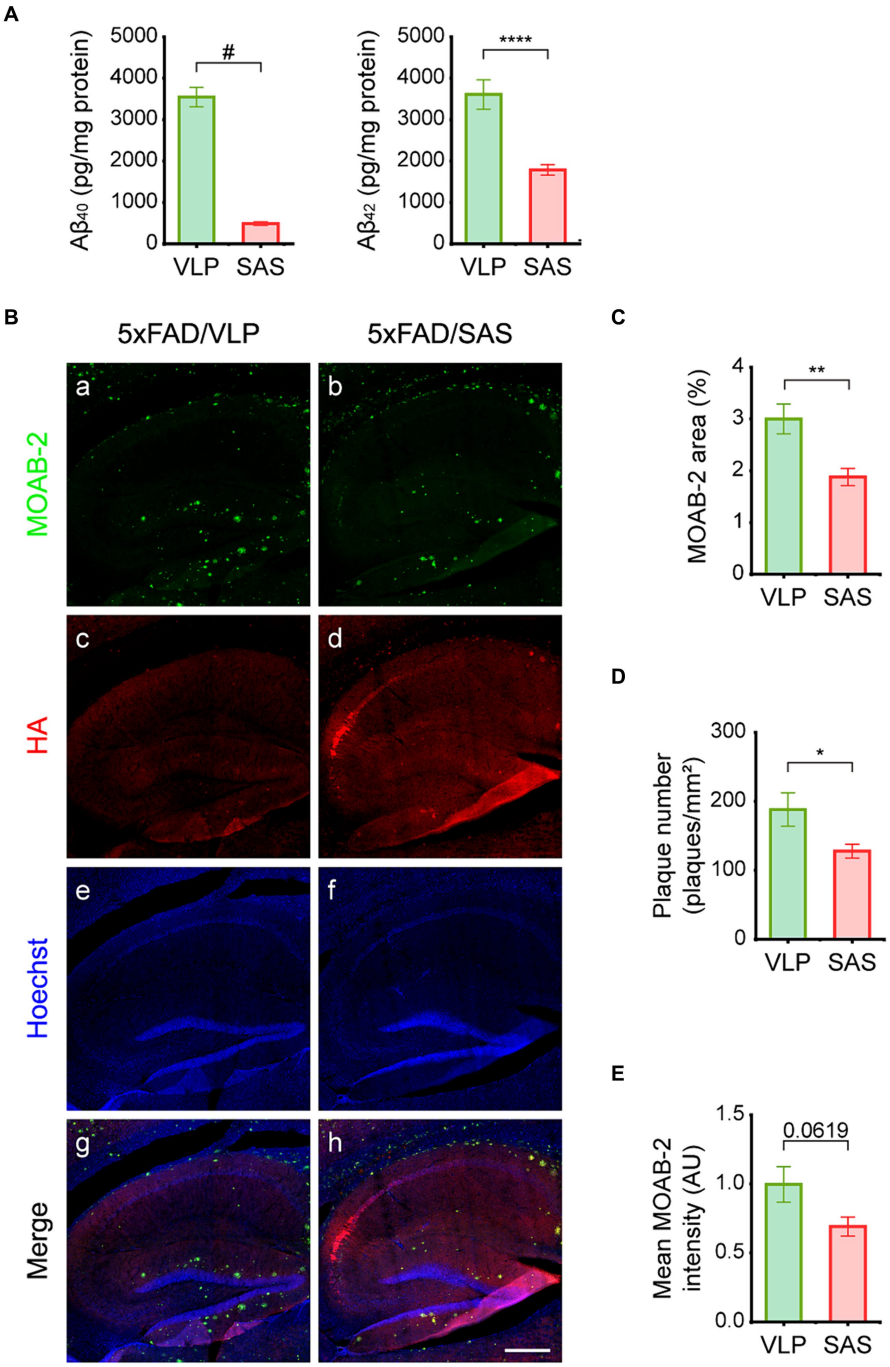
SAS reduces Aβ load in the 5xFAD mice. **(A)** Hippocampal extracts were analyzed by ELISA to detect Aβ_40_ and Aβ_42_. Aβ_40_ (Welch’s t-test: 5xFAD/VLP vs. 5xFAD/SAS, *p* < 0.0001) and Aβ_42_ (student’s *t*-test: 5xFAD/VLP vs. 5xFAD/SAS, *p* = 0.0006) were dramatically decreased by SAS expression. VLP, 5xFAD/VLP; SAS, 5xFAD/SAS. Male mice were used (5xFAD/VLP, *n* = 8, 5xFAD/SAS, *n* = 8). **(B)** Representative images of IHC of the 5xFAD mice injected with AAV9-VLP or AAV9-SAS using the MOAB-2 antibody and an anti-HA antibody. Scale bar, 300 μm. **(C–E)** Quantification of IHC results. **(C)** The MOAB-2 stained area (student’s *t*-test: 5xFAD/VLP vs. 5xFAD/SAS, *p* = 0.0093), **(D)** the number of plaques (student’s *t*-test: 5xFAD/VLP vs. 5xFAD/SAS, *p* = 0.0406), and **(E)** mean MOAB-2 intensity (student’s *t*-test: 5xFAD/VLP vs. 5xFAD/SAS, *p* = 0.0619) were calculated. ^*^p < 0.05, ^**^p < 0.01, ^****^p < 0.001, and ^#^*p* < 0.0005. Each bar and error bar represents the mean ± standard error of the mean (SEM). Male mice were used (5xFAD/VLP, *n* = 4, 5xFAD/SAS, *n* = 5).

The effects of SAS on Aβ burden were then examined. We employed an ELISA assay that can specifically detect full-length Aβ. The ELISA data revealed that the levels of soluble Aβ_40_ and Aβ_42_ were reduced by approximately 87 and 48%, respectively, in hippocampal lysates by SAS (Figure 6A). The reason Aβ_40_ is more profoundly reduced may be because it is more soluble than Aβ_42_, making it more accessible to SAS. Aβ plaques were also monitored by IHC using the MOAB-2 antibody (Figure 6B) that can specifically bind to Aβ but not to the full length or fragments of APP (Youmans et al., 2012). Both the area and number of plaques in IHC images were reduced by approximately 37 and 32%, respectively, by SAS (Figure 6B, panel b; Figures 6C,D). The intensity of plaques also appeared to be reduced, although the difference was not statistically significant (Figure 6E). Taken together, these results indicate that SAS reduced the levels of soluble Aβ and plaques in the 5xFAD mice.

### 3.7 SAS attenuates cognitive deficits in the 5xFAD mice

**Figure 7:**
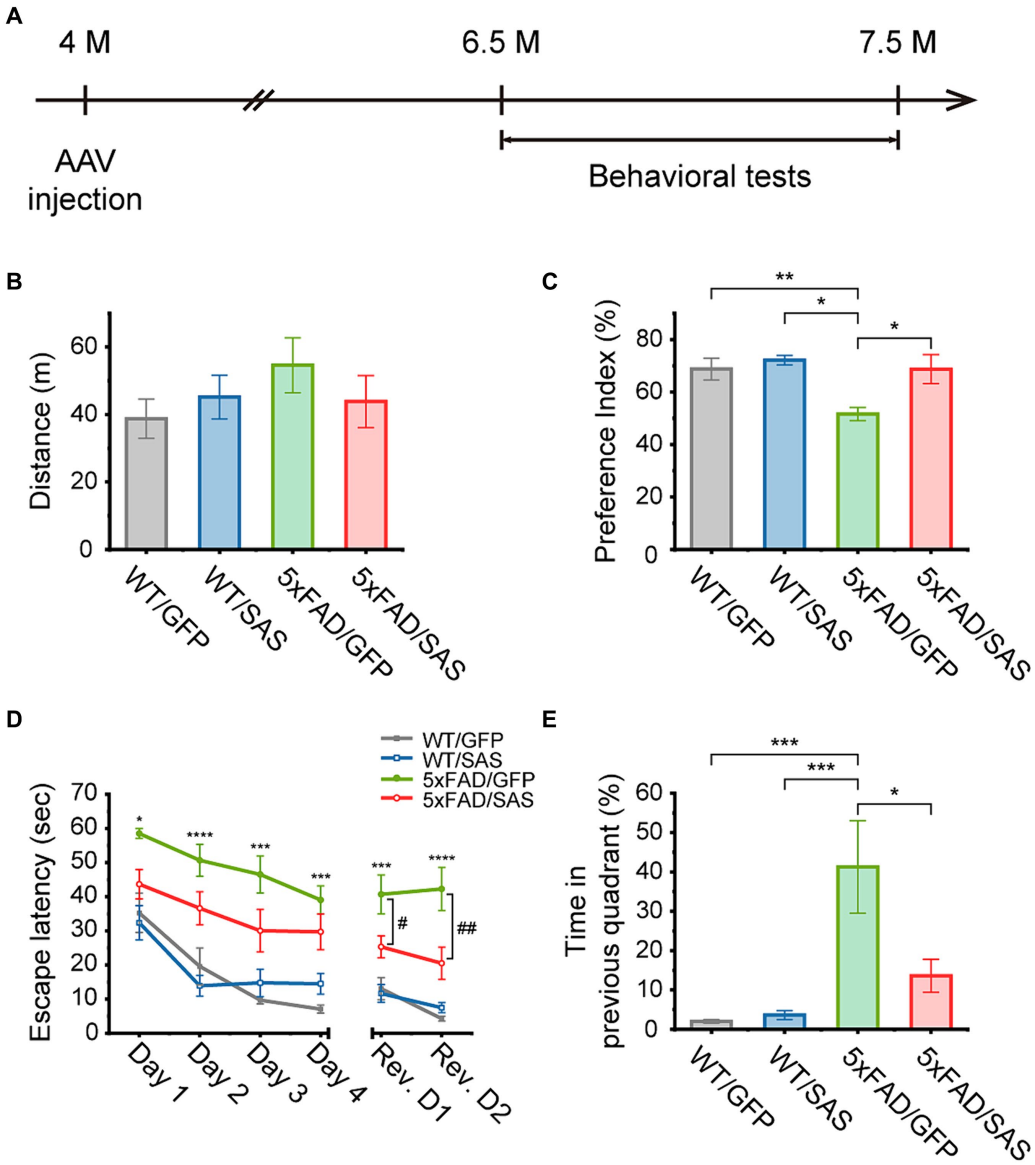
SAS attenuates cognitive deficits in the 5xFAD mice. **(A)** Experimental scheme of stereotaxic injection of AAV9 vectors and the behavioral tests. The mice were injected at the age of 4 months, and subjected to behavioral tests started at the age of 6.5 months. **(B)** An open-field test was performed to measure the traveled distance of each mouse to investigate the general locomotor activity of the animals. There was no significant difference in distance between all groups (one-way ANOVA: ns, no significance). **(C)** A novel object recognition (NOR) test was performed to measure the preference index for examination of recognition memory. The decreased preference index in 5xFAD/GFP were normalized in the 5xFAD/SAS group (one-way ANOVA followed by Tukey’s post-hoc test: WT/GFP vs. 5xFAD/GFP, *p* = 0.0290; WT/SAS vs. 5xFAD/GFP, *p* = 0.0094; 5xFAD/GFP vs. 5xFAD/SAS, *p* = 0.0438). **(D,E)** A Morris water maze (MWM) test was performed to explore the effect of SAS on spatial learning and cognitive flexibility. A daily training session (4 trials per session) during 4 consecutive days (Day 1–Day 4) were performed. The platform was removed 72 h after the last trial, and then cognitive flexibility was examined with a daily training session (4 trials per session) over 2 consecutive days (Rev. D1–Rev. D2). Rev. D, reversal training day. **(D)** Escape latency was measured to assess spatial learning and cognitive flexibility. Increased escape latency in 5xFAD/GFP was reduced in 5xFAD/SAS in initial training and reversal training (one-way ANOVA followed by Tukey’s post-hoc test: ^*^p < 0.05, ^***^p < 0.005, and ^****^p < 0.001 significant between WT/GFP and 5xFAD/GFP; ^#^*p* < 0.05, and ^##^*p* < 0.01), significant between 5xFAD/GFP and 5xFAD/SAS. **(E)** Time spent in the quadrant where the platform was previously located was measured during reversal session day 2 to examine cognitive flexibility. The increased time spent in the previous quadrant in 5xFAD/GFP was decreased in 5xFAD/SAS (one-way ANOVA followed by Tukey’s post-hoc test: WT/GFP vs. 5xFAD/GFP, *p* = 0.0008; WT/SAS vs. 5xFAD/GFP, *p* = 0.0009; 5xFAD/GFP vs. 5xFAD/SAS, *p* = 0.0121). Each bar or data point, and error bar represents the mean ± standard error of the mean (SEM). Male and female mice were used (WT/GFP, *n* = 6; WT/SAS, *n* = 7; 5xFAD/GFP, *n* = 6; 5xFAD/SAS, *n* = 8).

We then explored the effects of SAS on cognitive functions in the 5xFAD mice. The 5xFAD mice were injected with AAV9-SAS by stereotaxic delivery into the bilateral ventricles at 4 months of age and were analyzed by a series of behavioral tests starting at 6.5 months of age (Figure 7A). AAV9-GFP vectors were used as a negative control.

An open-field test (OFT) was performed to assess locomotor activity. There were no significant differences in the distance traveled among the WT and 5xFAD mice injected with either AAV9-GFP or AAV9-SAS (Figure 7B). These results indicate that SAS did not affect locomotor activity in mice.

In the novel object recognition (NOR) test, the 5xFAD mice did not spend more time exploring novel objects over familiar ones, while the WT mice spent more time exploring novel objects. Neither AAV9-GFP nor AAV9-SAS affected this behavior in the WT mice. However, AAV9-SAS, but not AAV9-GFP, completely normalized this behavioral deficit in the 5xFAS mice (Figure 7C). These data suggest that SAS can attenuate the impaired recognition memory in the 5xFAD mice.

In the Morris water maze (MWM) test, mice were trained to find a hidden platform for 4 consecutive days. During this training session, the 5xFAD mice exhibited severely impaired spatial learning abilities, as demonstrated by the longer time needed to locate the platform (escape latency), in comparison to the WT mice. Injection with AAV9-SAS appeared to improve the ability to locate the platform in the 5xFAD mice; however, the improvements were not statistically significant (Figure 7D, green vs. red). The platform was repositioned to the opposite quadrant 3 days after the final training session to evaluate the cognitive flexibility. During this reversal learning test, the 5xFAD mice located the repositioned platform less efficiently compared to the WT mice (Figure 7D, gray vs. green). Furthermore, they spent significantly more time in the quadrant where the hidden platform was previously positioned compared to the WT mice (Figure 7E). In contrast, the 5xFAD mice injected with AAV9-SAS located the repositioned platform more rapidly than their counterparts with statistical significance (Figure 7D, green vs. red) and spent significantly less time in the quadrant where the platform was previously positioned (Figure 7E). These results suggest that SAS attenuates the defects in spatial memory and cognition in the 5xFAD mice. Collectively, SAS expression by AAV9-SAS injection into the lateral ventricles attenuates the cognitive deficits in the 5xFAD mice.

## 4 Discussion

Our group previously reported that the NIa protease attenuated Aβ burden and cognitive impairments in Tg-APPswe/PS1dE9 mice (Han et al., 2010; Kim et al., 2012). Since the NIa protease is naturally located in the cytosol, our data supported the hypothesis that cytosolic Aβ is implicated in the pathogenesis of AD. As an extension of this previous study, we generated a fusion protein, SAS, to examine the physiological consequences when the NIa protease is located in intracellular luminal spaces such as the ER, Golgi, and endosomes (Figure 1). In designing SAS, we intriguingly utilized the transmembrane and intracellular domains of BACE1 so that SAS co-localizes with and counteracts the vicious activity of BACE1.

In *in vitro* experiments, we demonstrated that SAS can cleave the α-site in APP (Figure 2) and Aβ as expected (Figure 3). Similar events might occur *in vivo* and account for the beneficial effects of AAV9-SAS in the 5xFAD mice. The SAS-mediated cleavage of APP at the α-site may result in two major beneficial consequences: reduced production of Aβ and CTF-β, and increased production of sAPPα (Figures 5, 6).

1. BACE1 plays a pivotal role in the production of Aβ by cleaving APP at the β-site. BACE1 and APP are transported through separate secretory pathways in the Golgi (Burrinha and Guimas Almeida, 2022) and they converge mainly in endosomes (Wang et al., 2017; Burrinha and Guimas Almeida, 2022). The accumulation of APP in endosomes is abnormally enhanced in pathological conditions, leading to increased Aβ production (Burrinha and Guimas Almeida, 2022). We hypothesize that SAS competes with BACE1 in endosomes for APP processing and shifts the equilibrium from the amyloidogenic to the non-amyloidogenic pathway. CTF-β accumulation in lysosomes leads to poor acidification of lysosomes, resulting in abnormalities in the autophagy-lysosomal pathway, the primary pathway for the degradation of intracellular wastes in neurons (Mustaly-Kalimi et al., 2022). In the present study, we demonstrated that SAS reduced the levels of Aβ and CTF-β in the 5xFAD mice.
2. The α-cleavage product of APP, sAPPα, possesses a variety of beneficial functions in neurons. Numerous previous studies have demonstrated that sAPPα exerts neurotrophic and neuroprotective effects: it enhances long-term neuronal survival and neurite outgrowth (Araki et al., 1991), and it also protects neuronal cells from various insults such as hypoglycemic damage, glutamate excitotoxicity, reactive oxygen species, and most importantly, Aβ (Habib et al., 2017; Mockett et al., 2017; Dar and Glazner, 2020). Additionally, sAPPα acts as an enzyme inhibitor targeting BACE1 (Obregon et al., 2012) and glycogen synthase kinase-3β that is involved in tau hyperphosphorylation (Deng et al., 2013). Consequently, gene delivery-mediated expression of sAPPα attenuated AD pathologies in mice (Fol et al., 2016; Tan et al., 2018). Our data demonstrated that SAS increases the sAPPα level.
3. Exogenous Aβ is uptaken by neurons through endocytosis. The endocytosed Aβ impairs vesicular protein trafficking in endosomes (Marshall et al., 2020), and hampers protein degradation by inhibiting proteasome activity in endosomes and the cytosol (Almeida et al., 2006). The endocytosed Aβ can also reach the mitochondria and cause malfunctions (Cenini et al., 2016). Since SAS is located in endosomes, it can cleave the endocytosed Aβ en route from extracellular space to the cytosol, mitochondria, and even the nucleus.

In our lab, the 5xFAD mice exhibited significantly impaired cognitive functions when evaluated by NOR and MWM tests. We thus examined the therapeutic effects of AAV9-SAS in these categories. Intraventricular injection of AAV9-SAS completely normalized the defective recognition memory of the 5xFAD mice in the NOR test (Figure 7C). AAV9-SAS also improved learning ability during the training session in the MWM test, which, however, was not statistically significant (Figure 7D, *p*-values at days 1, 2, 3, 4 were 0.11, 0.15, 0.11, 0.38, respectively). To our surprise, AAV9-SAS significantly normalized the defective cognitive flexibility in the reversal learning session (Figures 7D,E). The reasons for the lack of statistical significance during the training session might be multiple: timing of viral injection and behavioral evaluation, doses and routes of viral injection, and number of the subjected mice. It is also intriguing to note that SAS is most intensively expressed in the CA2 region (Figure 4B; Supplementary Figure S2). It has been shown that the CA2 region is associated with cognitive flexibility in the similar MWM test (Caruana et al., 2012; Lehr et al., 2023). Therefore, a correlation between the expression of SAS and the consequential behavioral benefits appears to exist.

In summary, we showed that SAS possesses the proper beneficial characteristics as an α-secretase. In addition, SAS has strict substrate specificity cleaving APP and Aβ at the α-sites. We found a few other membrane or secreted proteins predicted to be efficiently cleaved by SAS (data not shown). On the contrary, endogenous α-secretases, including ADAM10, have broad substrate specificity, thus cleaving numerous cellular target proteins. With the recent advancement of gene therapy in the AD therapy realm, we propose that SAS can be a viable option for a gene therapy modality for AD. This study can also provide a platform for the development of novel drugs for AD.

## Data availability statement

The raw data supporting the conclusions of this article will be made available by the authors, without undue reservation.

## Ethics statement

The animal study was approved by Institutional Animal Care and Use Committee (IACUC) of Gwangju Institute of Science and Technology (GIST). The study was conducted in accordance with the local legislation and institutional requirements.

## Author contributions

SBK: Conceptualization, Data curation, Formal analysis, Investigation, Methodology, Visualization, Writing – original draft, Writing – review & editing. B-RM: Data curation, Formal analysis, Investigation, Writing – review & editing. SYK: Conceptualization, Investigation, Methodology, Writing – review & editing. ME: Investigation, Writing – review & editing. EJP: Investigation, Writing – review & editing. W-SC: Funding acquisition, Supervision, Writing – review & editing. WJP: Conceptualization, Funding acquisition, Supervision, Writing – original draft, Writing – review & editing.
