# Supplementary material for: Therapeutic effects of a novel synthetic α-secretase
Source: Front Aging Neurosci. 2024 Jun 7;16:1383905. doi: 10.3389/fnagi.2024.1383905 (PMC11191342; doi:10.3389/fnagi.2024.1383905)
Supplement: Supplementary file 1 [file Data_Sheet_1.docx]

Supplementary Material

Therapeutic Effects of a Novel Synthetic α-Secretase

Sung Bin Kim^1^, Bo-Ram Mun^2^, Sung Yoon Kim^1^, Muthukumar Elangovan^1^, Euy Jun Park^1^, Won-Seok Choi^2^, Woo Jin Park^1*^

*** Correspondence:** Woo Jin Park, woojinpark@icloud.com

| **Antibody** | **Host** | **Dilution** | **Source** | **Cat. No.** | **Target** |
| --- | --- | --- | --- | --- | --- |
| Myc (9B11) | Mouse monoclonal | WB 1:1000  ICC 1:1000 | CST | 2276S |  |
| Myc | Rabbit polyclonal | WB 1:1000 | CST | 2272S |  |
| HA (3F10) | Rat monoclonal | WB 1:1000  ICC 1:1000  IHC 1:1000 | Roche | 11867423001 |  |
| APP (LN27) | Mouse monoclonal | WB 1:1000 | Invitrogen | 13-0200 | N-terminus of APP |
| APP (Y188) | Rabbit monoclonal | WB 1:5000  IHC 1:500 | Abcam | ab32136 |  |
| APP, Aβ (6E10) | Mouse monoclonal | WB 1:1000 | Biolegend | 803001 | CTF-β of APP |
| BACE1 (EPR3956) | Rabbit monoclonal | WB 1:1000 | Abcam | ab108394 | BACE1 and SAS |
| ADAM10 | Rabbit polyclonal | WB 1:1000 | Abcam | Ab1997 |  |
| sAPPα (2B3) | Mouse monoclonal | WB 1:50 | IBL | 11088 | Specifically sAPPα, and *sAPPα’ |
| sAPPβ | Rabbit polyclonal | WB 1:1000 | Biolegend | 813401 | Specifically sAPPβ |
| Aβ (MOAB-2) | Mouse monoclonal | IHC 1:1000 | Novus Biologicals | NBP2-13075 | Specifically Aβ but not APP |
| Aβ_x-42_ (12F4) | Mouse monoclonal | ICC 1:250 | Biolegend | SIG-39142 | Specifically C-terminus of Aβ_42_ |
| α-tubulin | Mouse monoclonal | WB 1:1000 | Santa Cruz | sc-5286 |  |
| β-actin | Mouse monoclonal | WB 1:1000 | Santa Cruz | sc-47778 |  |
| GAPDH | Rabbit  monoclonal | WB 1:1000 | CST | 2118 |  |
| Anti-mouse 405 | Goat | ICC 1:1000 | Invitrogen | 31553 |  |
| Anti-mouse 488 | Goat | IHC 1:1000 | Invitrogen | 11001 |  |
| Anti-rabbit 488 | Goat | IHC 1:1000 | Invitrogen | 11008 |  |
| Anti-rat 555 | Goat | ICC 1:1000  IHC 1:1000 | Invitrogen | 21434 |  |
| Anti-mouse 555 | Goat | ICC 1:1000 | Invitrogen | 21422 |  |
| Anti-mouse HRP | Goat | WB 1:10000 | Invitrogen | 31430 |  |
| Anti-rabbit HRP | Goat | WB 1:10000 | Invitrogen | 31460 |  |
| Anti-rat HRP | Goat | WB 1:10000 | Invitrogen | 31470 |  |

**Supplementary Table 1** List of antibodies used in this study

*sAPPα’ is a soluble product of APP cleavage by SAS which lacks an amino acid (K16) at the C-terminus of sAPPα.


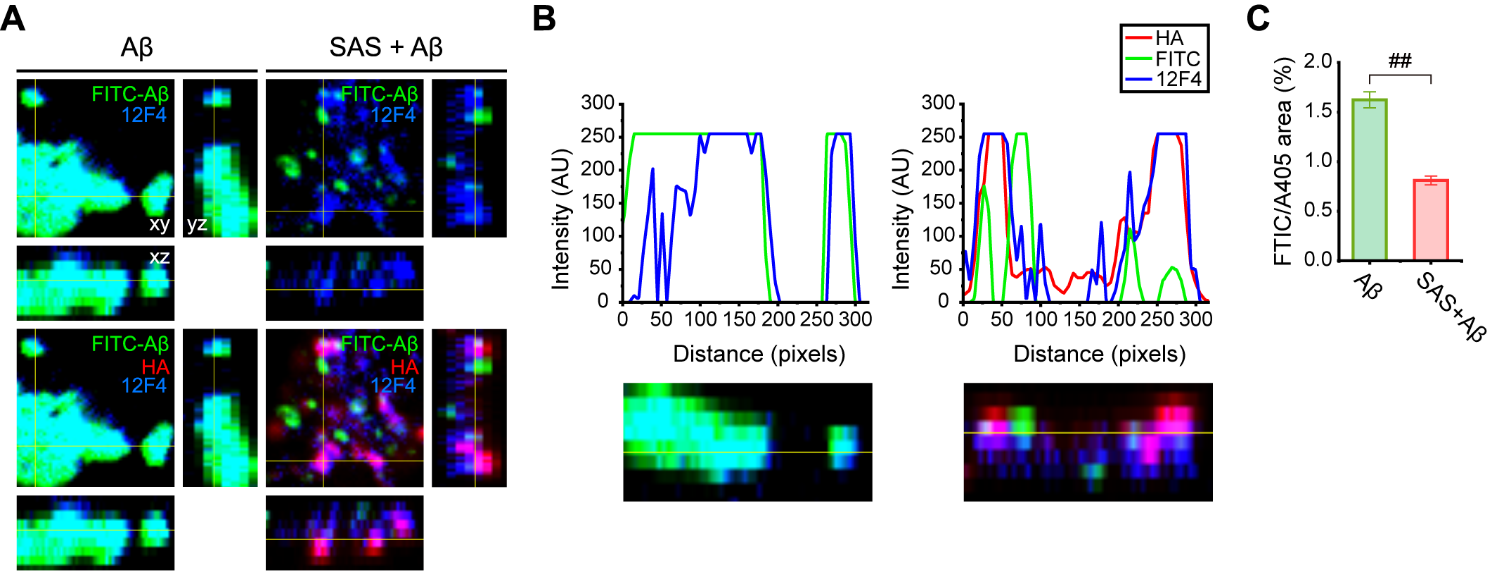


**Supplementary Figure 1** Co-localization analysis of N-/C-terminus of FITC-Aβ_42_. (A) The zoomed orthogonal images of Z-stack from Figure 3C are represented. The upper row shows the merge of green and blue, and the below shows the merge of green, red, and blue. The large image shows the xy view, the bottom image shows the xz and the right image shows the yz. Most of FITC, 12F4 signals were separated in the SAS expressing cells treated with FITC-Aβ_42_, whereas they were co-localized in the cells only treated with FITC-Aβ_42_. (B) Intensity line profiles of the yellow line indicated in the below images, which are the yz views of each of the three channels merged. (C) The FITC/A405 area (Student's t-test : Aβ vs SAS+Aβ, *p* < 0.0001) were calculated from images obtained by research slide scanner. ## *p* < 0.0001. Each bar and error bar represents the mean ± standard error of the mean (SEM).


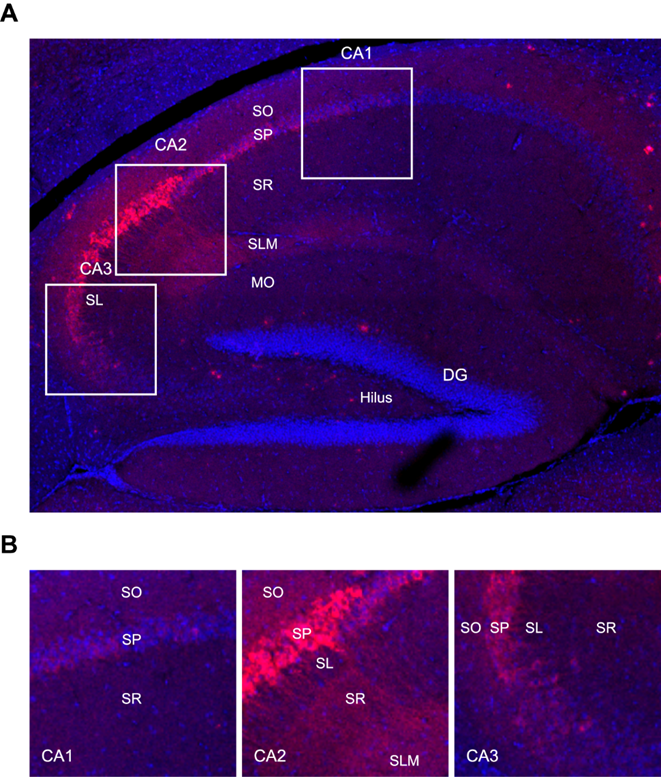


**Supplementary Figure 2** Detailed expression pattern of SAS in the hippocampus of 5xFAD mice. (A) The image from TG-SAS of Figure 4D is represented in a higher magnification. (B) The images of CA1-3 regions in the white lined open boxes are represented in a higher magnification. CA, Cornu Ammonis. DG, dentate gyrus. SO, stratum oriens. SP, stratum pyramidale. SR, stratum radiatum. SLM, stratum lacunosum moleculare. MO, stratum moleculare. SL, stratum lucidum.


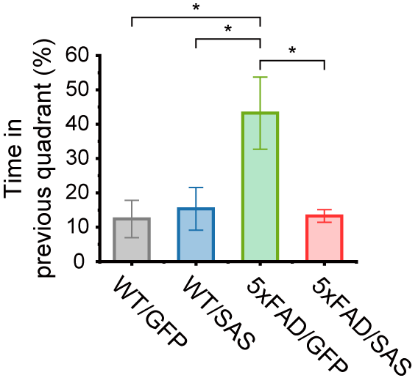


**Supplementary Figure 3** Time spent in the previous quadrant on reversal training day 1. Time spent in the previous target quadrant (%) on reversal day 1 of the Morris water maze test. (WT/GFP : *n* = 2 males, *n* = 4 females; WT/SAS : *n* = 3 males, *n* = 4 females; 5xFAD/GFP : *n* = 1 males, *n* = 5 females; 5xFAD/SAS : *n* = 1 male, *n* = 7 females). The data were analyzed statistically using a one-way ANOVA with post-hoc Tukey test. * *p* < 0.05. Each bar and error bar represents the mean ± standard error of the mean (SEM).


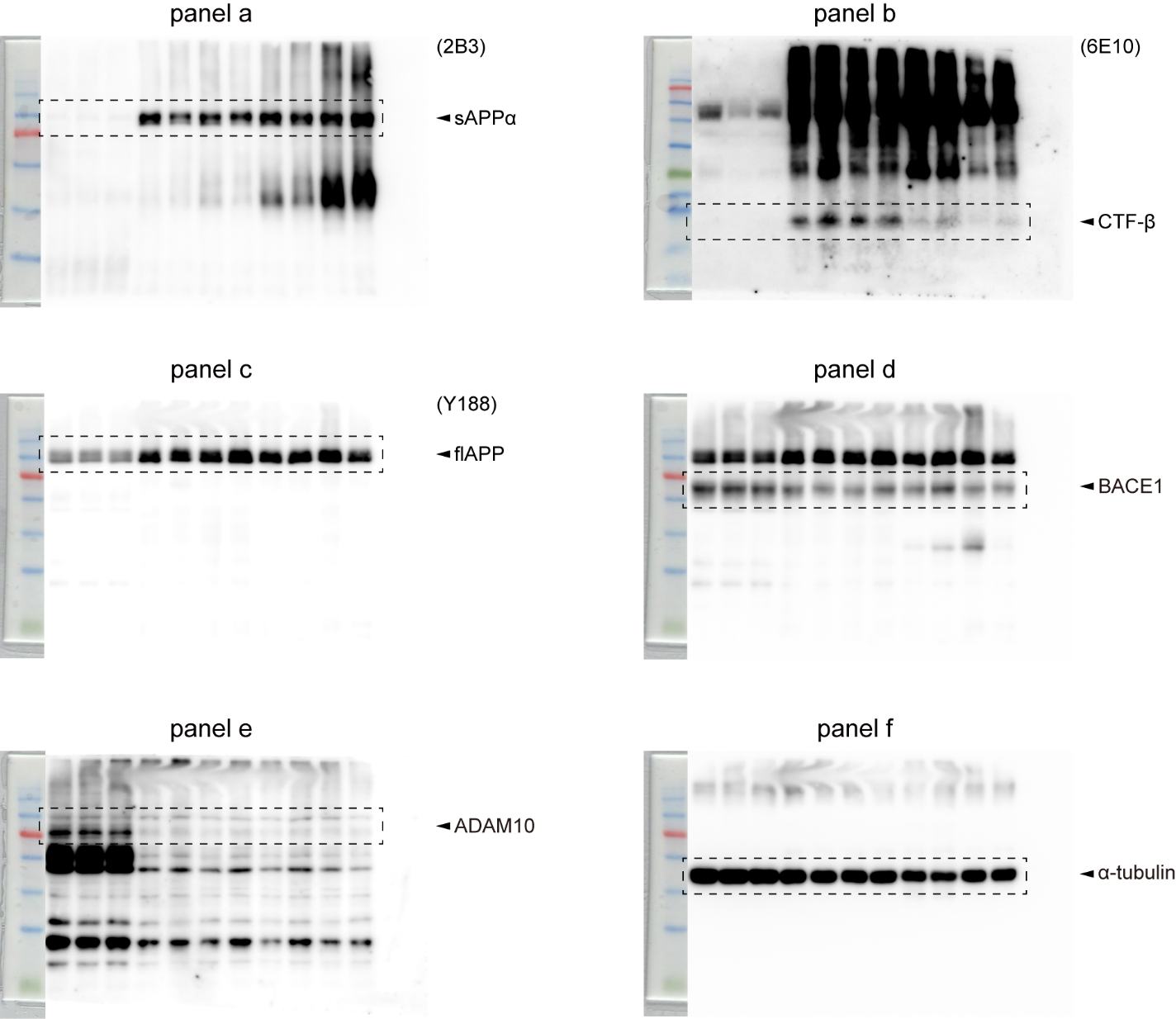


**Supplementary Figure 4** Uncropped western blot images of Figure 5A. Uncropped western blot images displayed in Figure 5A with molecular weight standard. The approximate extent of the cropped regions are represented by dotted line.
